# Grape Powder Intake Prevents Ovariectomy-Induced Anxiety-Like Behavior, Memory Impairment and High Blood Pressure in Female Wistar Rats

**DOI:** 10.1371/journal.pone.0074522

**Published:** 2013-09-09

**Authors:** Gaurav Patki, Farida H. Allam, Fatin Atrooz, An T. Dao, Naimesh Solanki, Gaurav Chugh, Mohammad Asghar, Faizan Jafri, Ritu Bohat, Karim A. Alkadhi, Samina Salim

**Affiliations:** Department of Pharmacological and Pharmaceutical Sciences, University of Houston, Houston, Texas, United States of America; University of Texas Medical Branch, United States of America

## Abstract

Diminished estrogen influence at menopause is reported to be associated with cognitive decline, heightened anxiety and hypertension. While estrogen therapy is often prescribed to overcome these behavioral and physiological deficits, antioxidants which have been shown beneficial are gaining nutritional intervention and popularity. Therefore, in the present study, utilizing the antioxidant properties of grapes, we have examined effect of 3 weeks of grape powder (GP; 15 g/L dissolved in tap water) treatment on anxiety-like behavior, learning-memory impairment and high blood pressure in ovariectomized (OVX) rats. Four groups of female Wistar rats were used; sham control, sham-GP treated, OVX and OVX+GP treated. We observed a significant increase in systolic and diastolic blood pressure in OVX rats as compared to sham-controls. Furthermore, ovariectomy increased anxiety-like behavior and caused learning and memory impairment in rats as compared to sham-controls. Interestingly, providing grape powder treated water to OVX rats restored both systolic and diastolic blood pressure, decreased anxiety-like behavior and improved memory function. Moreover, OVX rats exhibited an impaired long term potentiation which was restored with grape powder treatment. Furthermore, ovariectomy increased oxidative stress in the brain, serum and urine, selectively decreasing antioxidant enzyme, glyoxalase-1 protein expression in the hippocampus but not in the cortex and amygdala of OVX rats, while grape powder treatment reversed these effects. Other antioxidant enzyme levels, including manganese superoxide dismutase (SOD) and Cu/Zn SOD remained unchanged. We suggest that grape powder by regulating oxidative stress mechanisms exerts its protective effect on blood pressure, learning-memory and anxiety-like behavior. Our study is the first to examine behavioral, biochemical, physiological and electrophysiological outcome of estrogen depletion in rats and to test protective role of grape powder, all in the same study.

## Introduction

It is well known that estrogen is critical for normal brain function; and its depletion at menopause accounts, at least in part, for the cognitive decline, anxiety and hypertension [1]–[4]. In fact, estrogen is considered protective against anxiety [1]–[3], known to restore learning-memory function [1], [3] and also reported to help maintain normal blood pressure in females [2], [5].

Menopausal women, nearly 50 million in the United States alone, experience heightened anxiety, hypertension and memory impairment and often opt for estrogen replacement therapy [3], [6]. While this therapy offers some relief, it also carries with it the associated risks of breast cancer, heart disease and stroke [6]. In view of the present concern regarding the safety of estrogen replacement therapy, there is a growing demand for less invasive treatments to reduce and protect menopause-related conditions including hot flashes, weight gain, osteoporosis, fatigue, hypertension, anxiety and depression [1]–[4]. Reduction in estrogen levels during menopause is reported to be linked with elevated oxidative stress [7]–[9], which occurs due to an imbalance between production and elimination of reactive oxygen species (ROS) via the antioxidant defense system [10]. Relevant to this, dietary supplementation of antioxidants is reported to protect menopausal and postmenopausal women against high levels of oxidative stress [11]. Moreover, combined administration of vitamin C (ascorbic acid) and vitamin E (alpha-tocopherol) is reported to enhance protective action against ROS in mitochondrial membranes, which are rich in polyunsaturated fatty acids and quite vulnerable to oxidative stress [12], [13]. Recent research supports that mitochondrial damage and apoptosis caused by oxidative stress is prevented via both vitamin C and E [14]. The inclusion of B_6_ in micronutrient supplements in the diet has been shown to be important for the maintenance of reduced glutathione/oxidized glutathione ratio. Low ratio is an indicator of oxidative stress in cells and tissues. This is considered crucial for women of menopausal and postmenopausal age [15]. Also, the use of extracted phytoestrogens as dietary supplements by peri- and postmenopausal women has gained significance as an alternative to estrogen replacement therapy [16]. Consequently, use of herbal supplements, vitamins, minerals, antioxidants, and antidepressants is gaining attention [17]–[21].

Recently, we reported that grape powder treatment for 3 weeks prevented oxidative stress induced (via a pro-oxidant drug, BSO) anxiety-like behavior, learning-memory impairment and high blood pressure in rats [22]. Herein, we tested the antioxidant effect of grape powder in a more physiologically relevant model. The ovariectomy rodent model, in which ovaries are surgically removed resulting in depletion of the female hormone estrogen, is widely used to study menopause and menopause-associated conditions [23]. Using this model, we examined the effect of estrogen depletion on anxiety-like behavior, learning-memory function and blood pressure in the same set of female rats. More importantly, we examined whether grape powder treatment exerts a protective effect on anxiety-like behavior, learning-memory function and blood pressure in ovariectomized (OVX) female rats. The biochemical and electrophysiological basis for these effects also was examined. Our study is the first to examine behavioral, biochemical and electrophysiological outcome of estrogen depletion in rats and to test protective role of grape powder, all in the same study. Most studies have examined either the effect of ovariectomy on the behavioral [24]–[26], physiological [27]–[31], or biochemical level [31] or at the most have studied two aspects at one time [23].

## Materials and Methods

### Freeze Dried Grape Powder

Freeze dried grape powder was provided by the California Table Grape Commission in small sealed sachets. Upon receipt, the powder was stored at –80°C, and prepared fresh every day for feeding the rats by dissolving the powder in tap water at a concentration of 15 g/L [22]. As reported in our recent publication [22], this dose showed most pronounced effects on rat behavior [22]. The grape powder is a mixture of fresh red, green and blue-black California grapes (seeded and seedless varieties), freeze-dried and re-grounded, processed and stored to preserve the integrity of the biologically-active compounds. The powder contains resveratrol, flavans (including catechin), flavonols (including quercetin), anthocyanins and simple phenolics. Detailed composition and purity of this powder has been described in Allam et al. 2013 [22].

### Animals

All experiments were conducted in accordance with the NIH guidelines using approved protocols from the University of Houston Animal Care Committee. Ovariectomized (OVX) as well as sham operated (non-OVX) Wistar female rats were purchased from Charles River Laboratories. One set of Wistar female rats underwent ovariectomy (surgical removal of the ovaries) and the other set underwent mock surgery (opening of the abdominal cavity and sewing it back). Basically, a dorsal midline skin incision was made caudal to the posterior of the ribs. Using blunt dissection to tunnel subcutaneously, lateral to the skin incision, the muscles of the posterior abdominal wall were separated in order to expose the abdominal cavity. The ovary is located in a fat pad beneath the muscles. The periovarian fat was grasped to lift and exteriorize the ovary. The fallopian tube was crushed and the ovary was removed by cutting above the clamped area. The skin incision was closed using wound clips. Charles River’s institutional Animal Care and Use Committee (IACUC) oversee the entire surgical process, including post-operative care prior to shipment. Rats upon arrival were acclimatized for one week before any treatment and provided with rat chow and drinking water *ad libitum*. PicoLab rodent diet 20 (cat# 5056) containing a mixture of 20% crude protein, 4.5% crude fat, 6% fiber and 7% minerals was purchased from LabDiet Inc. (Brentwood, MO, USA). Rats upon arrival were divided into four groups. Groups 1 and 2 (n = 10 rats/group) that underwent mock surgery (non- OVX) were considered as sham controls with one provided with tap water and the other group provided with grape powder treated tap water. Groups 3 and 4 that underwent ovariectomization surgery (OVX), were either provided with grape powder treated tap water or tap water alone for 3 weeks.

Twenty four hours after the last day of grape powder (GP) treatment, memory and anxiety-like behavior tests were conducted. Next, rats were subjected to blood pressure measurement, at the completion of which, rats were quickly decapitated, blood, urine and brain tissues collected and indices of oxidative stress measured as previously described by Salim et al. 2010 [32]–[34]. Separate set of rats were utilized for electrophysiology experiments.

### Anxiety Behavior tests

#### Open Field (OF) activity

Rats were placed in the center of the OF (60×40 cm) and left free to explore the arena for 15 min and movement quantified using Opto-Varimex Micro Activity Meter v2.00 system (Optomax, Columbus Instruments; OH) as previously described in Salim et al. 2010 [32]–[34]. Total time spent in the center of the arena, rearings and fecal boli were calculated.

#### Light-Dark (LD) exploration

The light-dark box consisted of a light and a dark compartment separated with a single opening for passage from one compartment to the other and total time spent in the lit area was recorded [34]. Less time spent in light is considered as a measure of anxiety-like behavior. A total of *n* = 10 rats/group were used for these experiments.

### Learning and memory tests

Radial Arm Water Maze (RAWM): After ∼2h of anxiety tests, rats were subjected to learning and memory tests as published [22] using a black circular water filled pool with six swim paths.

Briefly, the rats were subjected to the first set of six learning trials (trials # 1–6) followed by a five min rest period and then another set of six learning trials (trials # 7-12) and tested for short-term memory 30 min after the end of 12^th^ trial. The rats were returned to their home cages and 24h later subjected to long-term memory test. A total of *n* = 10 rats/group were used for these experiments.

### Blood pressure measurement

Blood pressure measurement was done as previously described [35]. Briefly, rats were anesthetized with Inactin (100 mg/kg i.p.). Tracheotomy was performed to facilitate breathing. To measure blood pressure and collect blood samples, the left carotid artery was catheterized with PE-50 tubing. This tubing was connected to a pressure transducer, which was connected to an amplifier (GRASS, LP122). Blood pressure was continuously recorded for 30 min using GRASS PolyView Data Acquisition and Analysis Software systems (Astro-Med, GRASS Instrument Division, West Warwick, RI). After blood pressure measurement, animals were sacrificed and aliquots of blood and urine were withdrawn and plasma was isolated by centrifugation and kept at –80°C frozen until further use. A total of *n* = 8–10 rats/group were used for this set of experiments.

### In vivo electrophysiological recording

At the end of grape powder treatment, *in vivo* electrophysiological recordings from the dentate gyrus (DG) of the hippocampus was done as described [36], [37]. Rats were anesthetized via i.p. injections of urethane (1.2 g/kg, Sigma Aldrich, USA) and the rat head was fixed on a stereotaxic frame. On the right side of the brain, a hole was drilled for placing the concentric bipolar stimulation electrode which stimulates the perforant pathway via the angular bundle (AP: -8, L: 4.7, V: 1.2). The capillary glass (1–5 MΩ) recording electrode, filled with 2 M NaCl dye solution, was positioned to record the granular cell layer of the DG area of the right hippocampus (AP: -3, L: 2, V: 3.5). A 30 minute stabilization period (no stimulation) was allowed after the maximal response was found. A baseline was recorded for 20 minutes by giving test stimuli (30% of maximal, 1 pulse/30s). Early long-term potentiation (E-LTP) of the perforant pathway was evoked by a single train of high frequency stimulation (HFS) by delivering 8 pulses of 400 Hz every 10 seconds, repeated 8 times. The changes in the field excitatory post-synaptic potential (fEPSP) slope and population spike amplitude after 1 hour recording represent the E-LTP. The fEPSP slope represents synaptic strength while the pspike amplitude indicates the number of neurons reaching firing threshold [36], [37]. A total of *n* = 3–4 rats/group were used for this set of experiments.

### Brain Dissections and Preparation of Homogenates

Rats were anesthetized using mild anesthesia (Isoflurane, #57319-479-06, Phoenix Pharmaceuticals) immediately after behavior tests. The brains were quickly removed and rapidly frozen at –80°C until analysis. The hippocampus, amygdala and cortex were identified according to Paxinos and Watson [38] and grossly dissected out, homogenized and the protein concentration determined as described by Vollert et al. 2012 [32]–[34].

### Western Blot Analysis

Homogenates were subjected to SDS-polyacrylamide gel electrophoresis (PAGE) and western blotting. The following dilutions were used for detection of specific proteins. Glyoxalase (GLO)-1 (1∶200 dilution), Mn SOD (1∶1000 dilution) and Cu/Zn SOD (1∶1000 dilution), BDNF (1∶1000 dilution) and loading control (β-actin 1∶1000 dilution). Anti-rabbit horseradish peroxidase (HRP)-conjugated (1∶1000) or anti-mouse HRP-linked secondary antibody (1∶1000) were used as needed. Intensity of each immunoreactive band on the immunoblots (normalized to the β-actin loading control) was determined using Alpha Ease FC 4.0 (Alpha Innotech Corp., San Leandro, CA). A total of *n* = 3 rats/group were used for this set of experiments.

### Indices of oxidative stress

8-isoprostane levels in serum and urine were measured using EIA kit (Cayman, Ann Arbor, MI). Isoprostanes are a family of eicosanoids of non-enzymatic origin produced by the random oxidation of tissue phospholipids by oxygen radicals [33]. The OxyBlot™ Protein Oxidation Detection Kit (EMD Millipore Corp. #S7150) was used for immunoblot detection of carbonyl groups introduced into proteins by oxidative reactions. Basically, equal amount (20 µg) of protein homogenate from different brain regions (prepared as indicated above) were subjected to this kit based reaction following manufacturer’s instructions, which allows detection of carbonylation of proteins in the homogenates using western blotting method. Protein-nitration was determined as described by Salim et al. [32], [33]. Briefly, equal amounts of protein (25 µg per lane) were loaded on a 10% SDS-PAGE and subjected to immunoblotting with HRP-conjugated nitrotyrosine antibody (EMD Millipore Corp) at a dilution of 1∶1000 in 2.5% BSA–TBS Tween solution. The blot was incubated for 1 h at room temperature while rocking, washed three times with 1% TBS-T solution for 30 min. The immunoreactive bands were developed using chemiluminescence reagent. A total of *n* = 10 rats/group were used for this set of experiments.

### Statistical Methods and Data Analysis

Data are expressed as mean ± SEM. Significance was determined by two way ANOVA followed by Bonferroni’s post-hoc test (GraphPad Software, Inc. San Diego, CA). A value of *P*< 0.05 was considered significant. Outliers where present were determined using the Grubbs test for outliers in the GraphPad Prism program. No data transformation was required.

## Results

### General parameters

**Figure 1:**
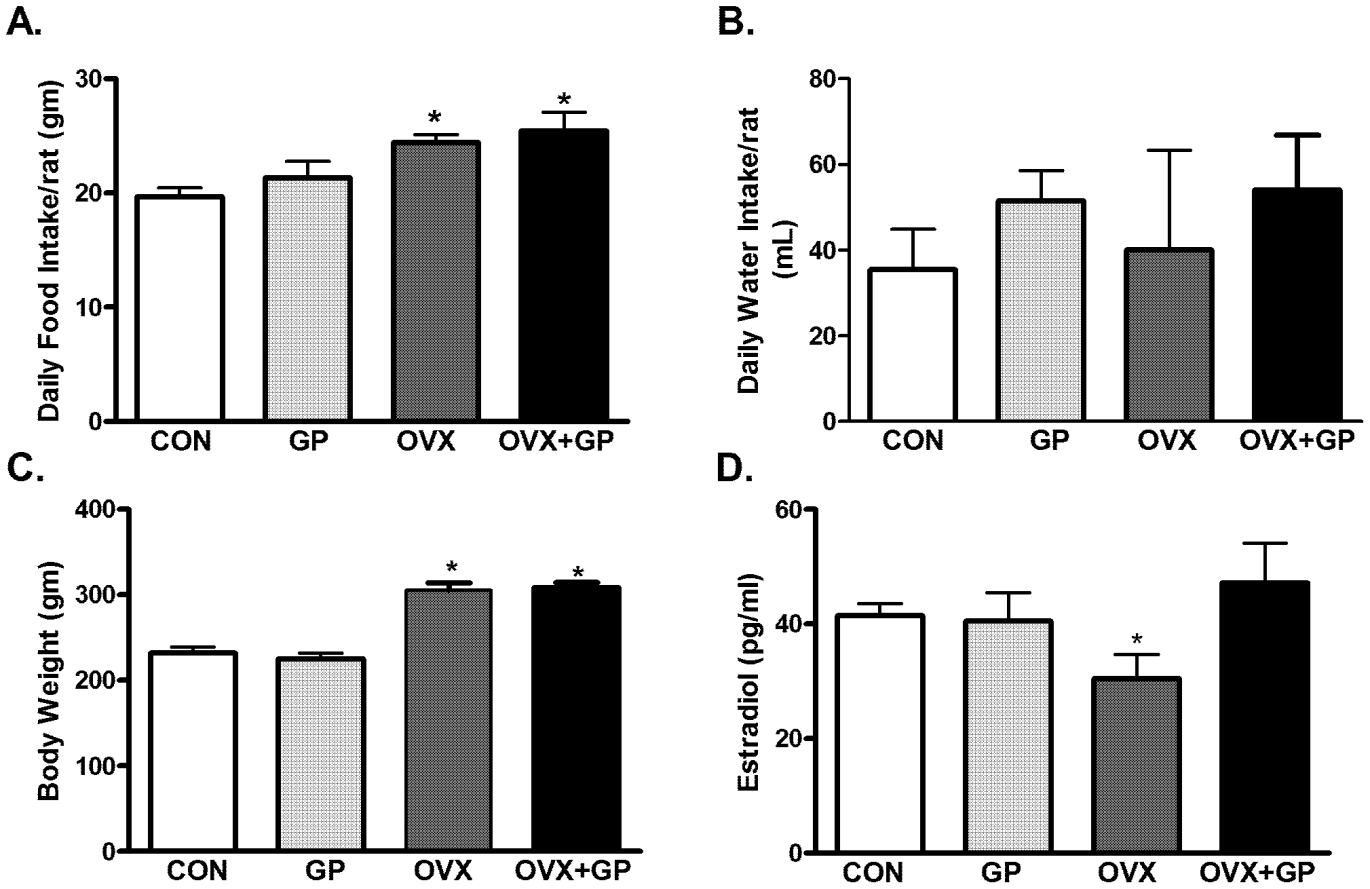
General body parameters. Four groups of female Wistar rats were utilized in this study, control (sham surgery) rats provided with tap water for drinking or grape powder (GP) treated tap water, ovariectomized rats (surgical removal of ovaries, OVX) provided with drinking water, or with GP treated tap water. Daily food intake (A) water intake (B) body weight (C) was measured in all groups. Estradiol levels were measured in plasma using Estradiol EIA Kit (D). Bars are means ± SEM, *n* = 10 rats/group, *P*<0.05, (*) significantly different from control and GP.

Daily food and water intake was measured in all rats. While sham- controls provided with tap water (CON: 19±1.42 g/day) or GP treated tap water (GP: 21±0.76 g/day) consumed similar amount of food per day, OVX (24±0.66 g/day) and OVX+GP (25±1.60 g/day) rats consumed significantly greater food/day **(** **Fig.1** **A)**. Water intake on the other hand remained unchanged in all groups with each group consuming 35–54 ml water per day **(** **Fig.1** **B)**. Body weight of OVX (305±8.8 g) and OVX+GP (308±6.0 g) groups was significantly higher than sham-controls provided with tap water (CON: 231±1.42 g) or GP treated tap water (GP: 225±0.76 g) **(** **Fig.1** **C)**. Plasma estradiol levels examined using a kit based assay, showed a significant decrease in OVX group (30.4±4.1 pg/ml) as compared to CON (41.5±2.1 pg/ml), GP (40.5±4.9 pg/ml) or OVX+GP groups (47.0±6.9 pg/ml) **(** **Fig.1** **D)**.

### Indices of oxidative stress

**Figure 2:**
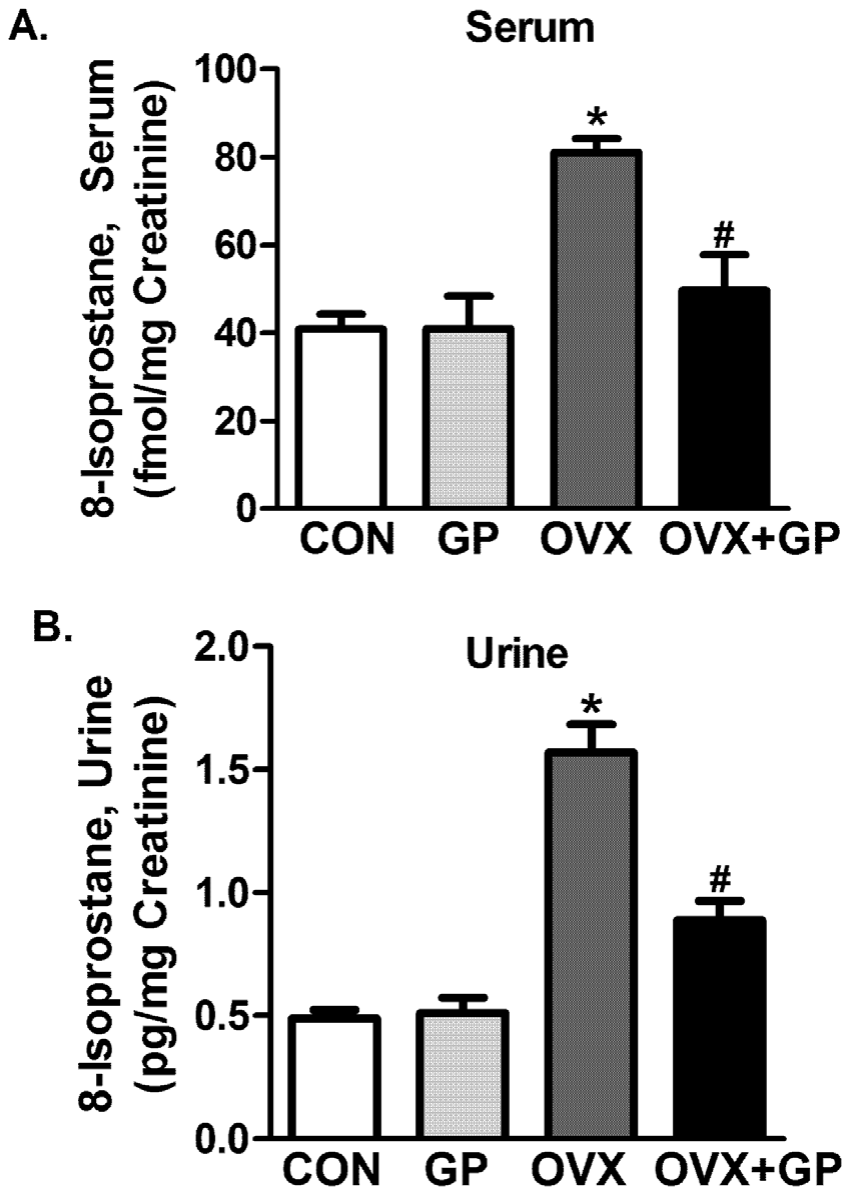
Analysis of marker of oxidative stress 8-isoprostane in CON, GP, OVX and OVX+GP rats. 8-isoprostane was measured in serum (A) and urine (B) using EIA kit. Bars are means ± SEM, *n* = 10 rats/group, *P*<0.05, (*) significantly different from control and GP and (#) significantly different from OVX. Group designations: CON (control; sham operated rats provided with tap water for drinking), GP (grape powder: sham operated rats provided with GP treated tap water for drinking), OVX (ovariectomized: rats subjected to surgical removal of ovaries and provided with tap water for drinking), OVX+GP (rats subjected to surgical removal of ovaries and provided with GP treated tap water for drinking).

**Figure 3:**
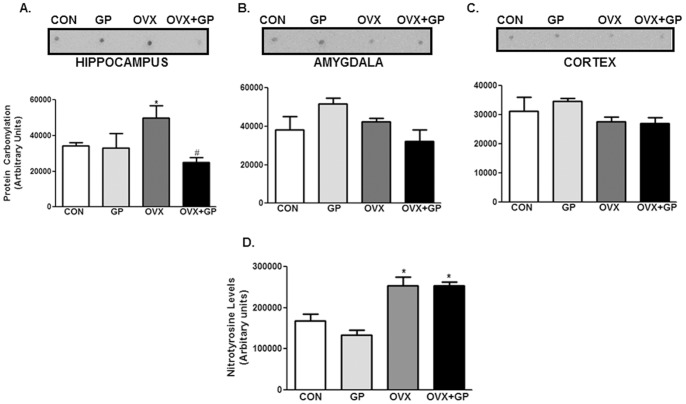
Analysis of protein carbonylation and nitrotyrosinylation in CON, GP, OVX and OVX+GP rats. The OxyBlot™ Protein Oxidation Detection Kit was used for immunoblot detection of carbonyl. Equal amount (20 µg) of protein from different brain regions were subjected to this kit based reaction by following manufacturer’s instructions. Brain homogenates from hippocampus were subjected to western blotting using anti-nitrotyrosine antibody. Bars are means ± SEM, *n* = 10 rats/group, *P*<0.05, (*) significantly different from control and GP. Group designations: CON (control; sham operated rats provided with tap water for drinking), GP (grape powder: sham operated rats provided with GP treated tap water for drinking), OVX (ovariectomized: rats subjected to surgical removal of ovaries and provided with tap water for drinking), OVX+GP (rats subjected to surgical removal of ovaries and provided with GP treated tap water for drinking).

Serum and urinary 8-isoprostane levels were significantly greater in OVX group as compared to sham-control (CON or GP) groups **(** **Fig.2** **A, B)**. Mean serum and urinary 8-isoprostane levels in GP group were not significantly different from CON. Protein carbonylation levels (marker of protein oxidation), also were significantly greater in OVX group in hippocampus **(** **Fig. 3A** **)** but not in amygdala **(** **Fig. 3B** **)** or cortex **(** **Fig. 3C** **)** as compared to the CON, GP or OVX+GP groups. Protein carbonylation levels were only significantly altered in hippocampus, hence, nitrotyrosine levels were estimated in the hippocampus and a significant increase in nitrotyrosine levels was observed in the ovariectomized groups (OVX alone and OVX+GP), surprisingly, GP had no effect on nitrotyrosine levels **(** **Fig. 3D** **)**.

### Anxiety-like behavior tests

**Figure 4:**
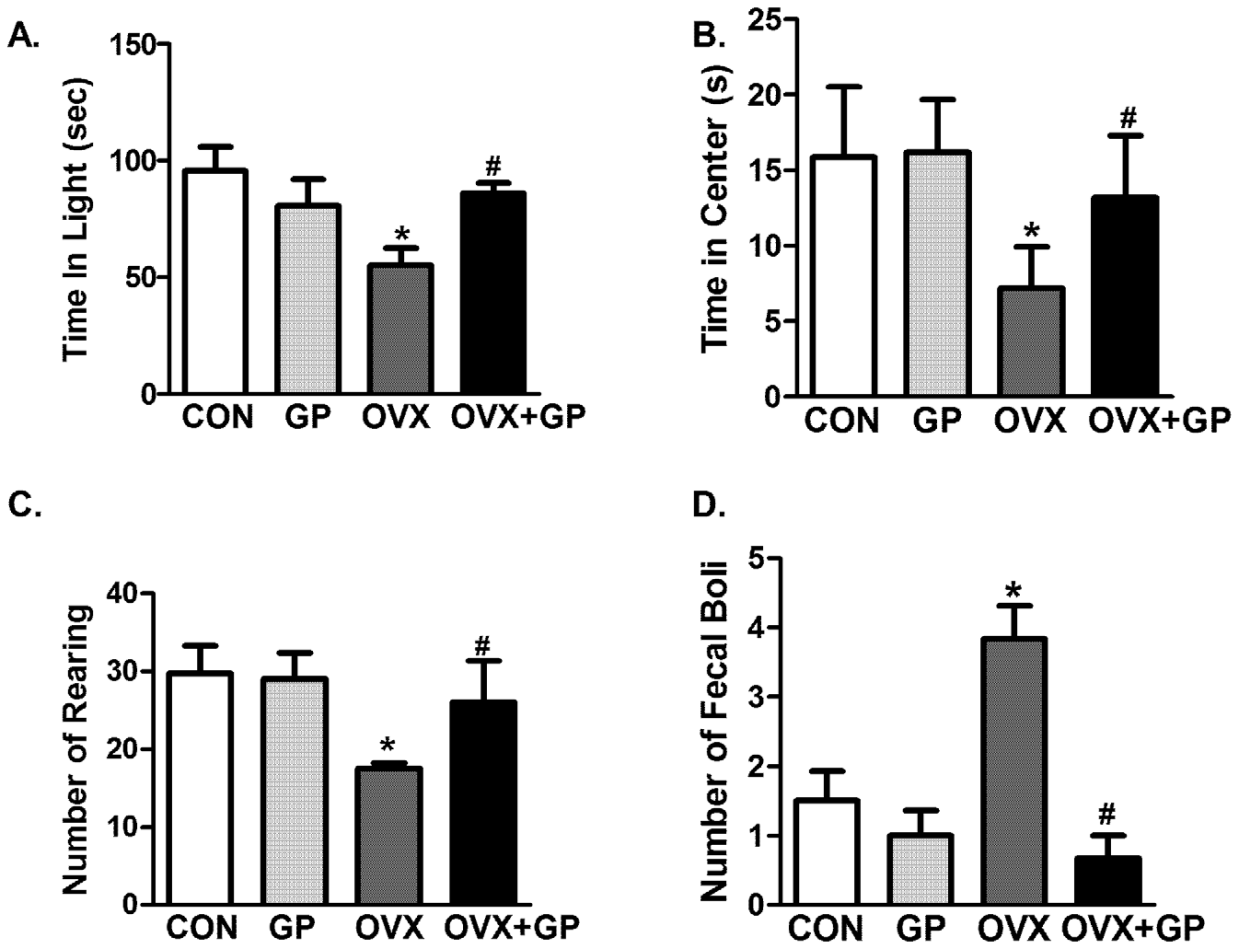
Examination of anxiety-like behavior tests including light-dark and open-field tests in CON, GP, OVX and OVX+GP rats. Time spent in the lighted area in the light–dark test (A) The open-field test determined center time (B), rearing (C) and fecal boli (D). Bars are means ± SEM, *n* = 10 rats/group, *P*<0.05, (*) significantly different from control and GP and (#) significantly different from OVX. Group designations: CON (control; sham operated rats provided with tap water for drinking), GP (grape powder: sham operated rats provided with GP treated tap water for drinking), OVX (ovariectomized: rats subjected to surgical removal of ovaries and provided with tap water for drinking), OVX+GP (rats subjected to surgical removal of ovaries and provided with GP treated tap water for drinking).

In the light-dark anxiety test, OVX rats spent significantly less time in the light compartment as compared to CON, GP and OVX+GP rats **(** **Fig. 4A** **)**. In the open field test, OVX rats spent significantly less time in the center of the open field as compared to CON, GP and OVX+GP rats **(** **Fig. 4B** **)**. Rearings recorded by the optomax software in the open-field test indicate that OVX rats displayed significantly reduced rearing as compared to CON, GP and OVX+GP rats **(** **Fig. 4C** **)**. Reduced rearing is indicative of increased anxiety. Fecal boli counts suggest that OVX rats had significantly increased number of fecal boli as compared to CON, GP or OVX+GP rats **(** **Fig. 4D** **)**, which indicates heightened anxiety in rodents. In all four parameters **(** **Fig. 4A-D** **)**, OVX+GP group exhibited behaviors similar to controls.

### Memory impairment

**Figure 5:**
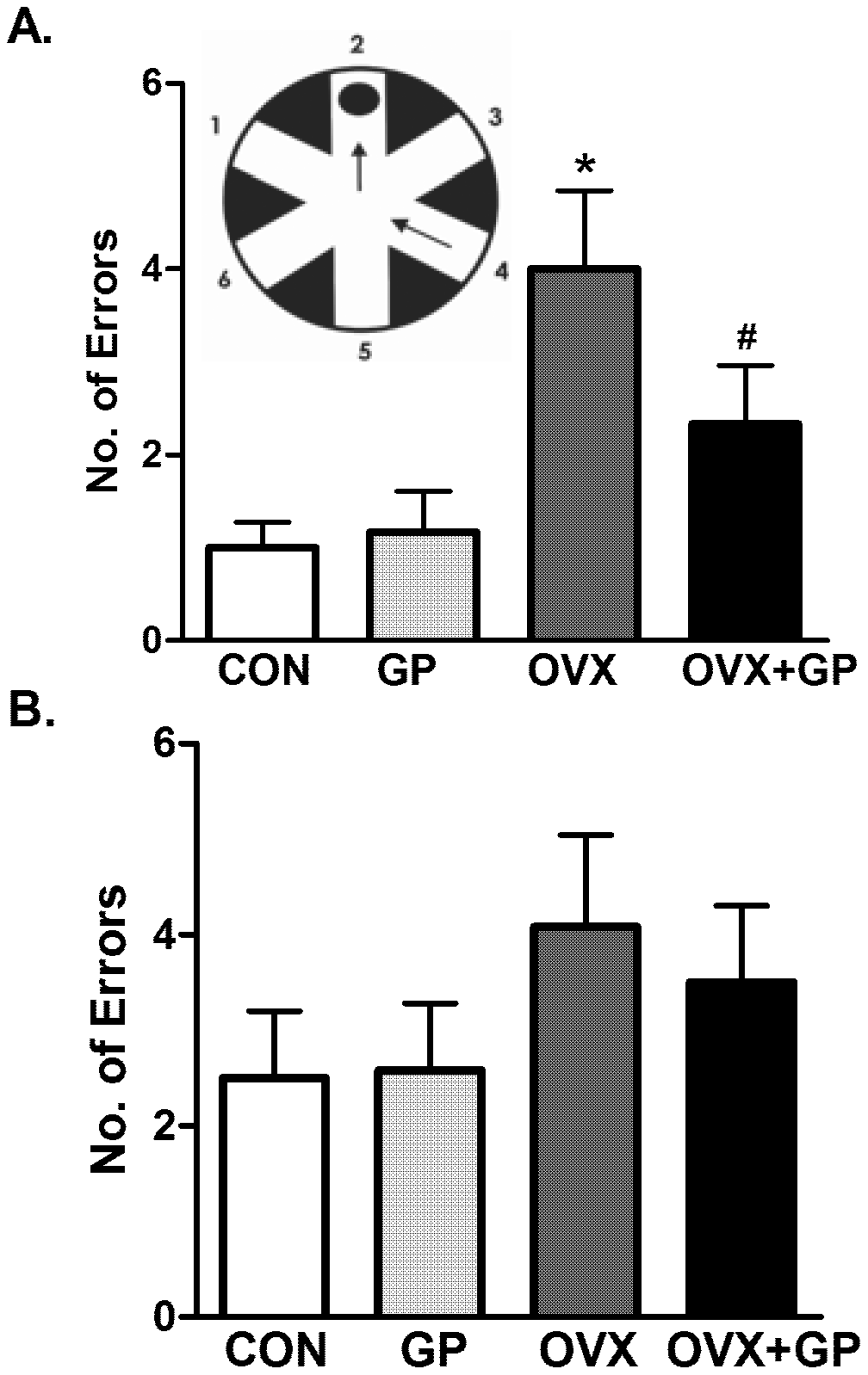
Radial arm water maze (RAWM) memory tests in CON, GP, OVX and OVX+GP rats. Short term (A) and long term (B) memory was assessed after a series of twelve radial arm water maze trials. The RAWM apparatus is shown as an insert containing a circular water pool with six swim paths. Bars are means ± SEM, *n* = 10 rats/group, *P*<0.05, (*) significantly different from control and GP. Group designations: CON (control; sham operated rats provided with tap water for drinking), GP (grape powder: sham operated rats provided with GP treated tap water for drinking), OVX (ovariectomized: rats subjected to surgical removal of ovaries and provided with tap water for drinking), OVX+GP (rats subjected to surgical removal of ovaries and provided with GP treated tap water for drinking).

OVX rats exhibited significantly higher number of errors in the STM test as compared to CON, GP, and OVX+GP rats **(** **Fig. 5A** **)**. However, in the LTM, OVX rats showed no significant effect when compared to CON, GP, and OVX+GP rats **(** **Fig. 5B** **)**. GP rats exhibited similar learning, short-term and long-term memory performance equivalent to control rats, indicating that grape powder treatment, alone, had no effect on long-term learning or short-term memory performance, compared to control rats **(** **Fig. 5A, B** **)**. Thus, under our experimental conditions, although grape powder did not significantly affect learning and memory in normal rats, it prevented OVX-induced impairment of STM.

### The impairment of E-LTP in the dentate gyrus (DG) caused by ovariectomy was rescued by grape powder (GP) treatment

**Figure 6:**
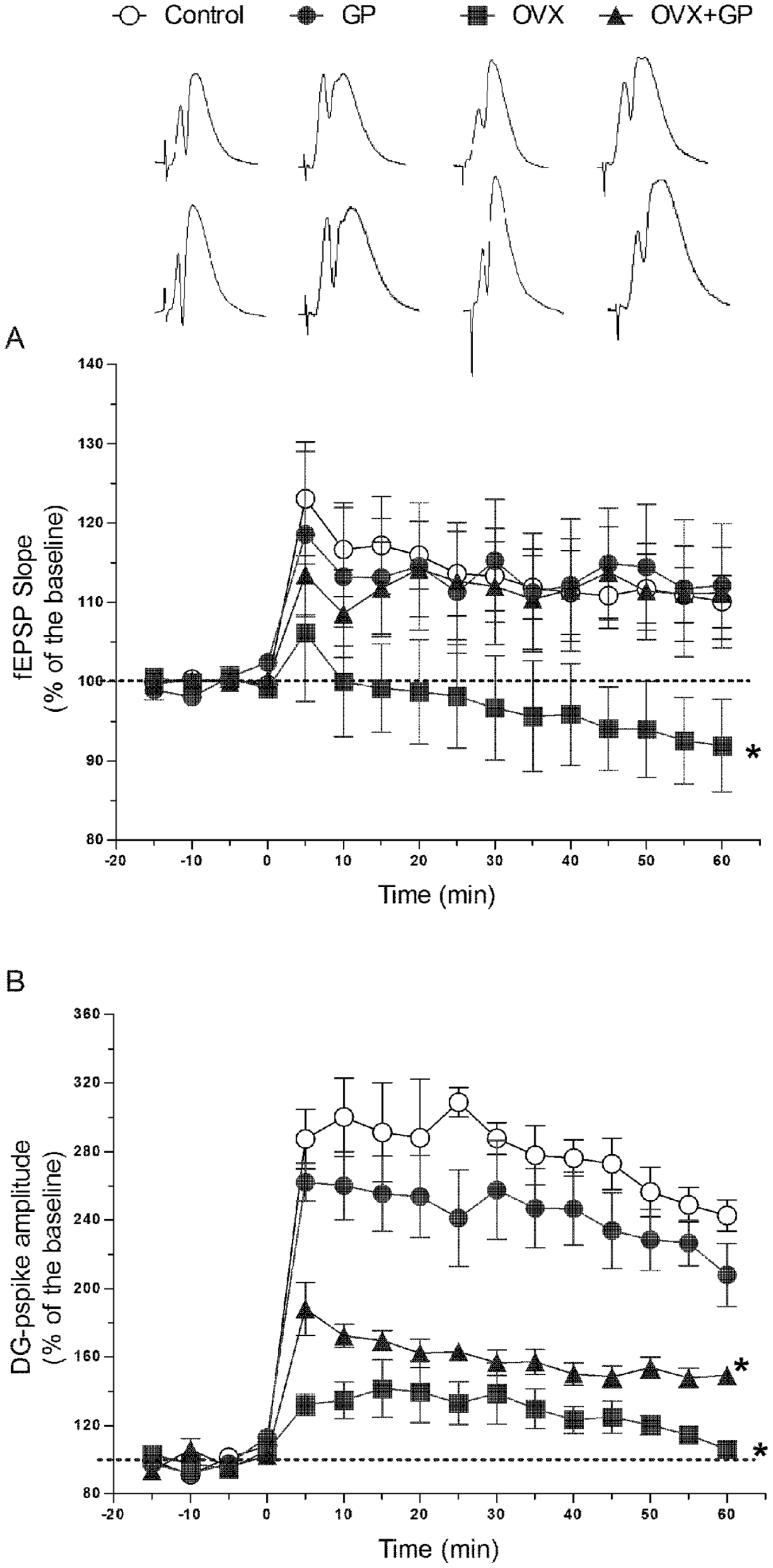
Long-term potentiation (LTP) in CON, GP, OVX and OVX+GP rats. E-LTP evoked by a single train of HFS, was measured in the dentate gyrus (DG) as changes in the fEPSP slope (A) and pspike amplitude (B). Ovariectomized rats exhibited significantly lower values of both fEPSP slope and pspike amplitude compared to those of other groups. However, LTP of OVX+GP rats were similar to those of control rats as measured by fEPSP. Each point is the mean ± SEM of 3–4 rats. (*) indicate significant difference from all other groups. Group designations: CON (control; sham operated rats provided with tap water for drinking), GP (grape powder: sham operated rats provided with GP treated tap water for drinking), OVX (ovariectomized: rats subjected to surgical removal of ovaries and provided with tap water for drinking), OVX+GP (rats subjected to surgical removal of ovaries and provided with GP treated tap water for drinking).

The changes of synaptic plasticity were assessed utilizing LTP measurement, the closest cellular analog of learning and memory. One hour after LTP induction, the fEPSP slope from the ovariectomized rats was significantly lower compared to all other groups (control: 110.098% ± 3.293, OVX+GP: 115.562%±5.33, GP: 112.1%±7.848, OVX: 91.93%±5.825) **(** **Fig.6A** **)**. Thus, ovariectomized rats exhibited an impaired E-LTP which was prevented by grape powder treatment as the fEPSP slope of OVX+GP rats was similar to that of control rats. Interestingly, the perforant synapses seemed suppressed throughout the 1 hour recording period. Also compared to baseline, at 1 hour after HFS the pspike amplitude of OVX rats (105.978%±3.435) was markedly lower than other groups (control: 242.585%±9.128, OVX+GP: 149.284%±3.257, GP: 207.943%±18.327) **(** **Fig.6B** **)**. Together, these results indicate that grape powder treatment reversed ovariectomy-induced inhibition of the perforant pathway synapses of the dentate gyrus (DG).

### Blood pressure measurement

**Figure 7:**
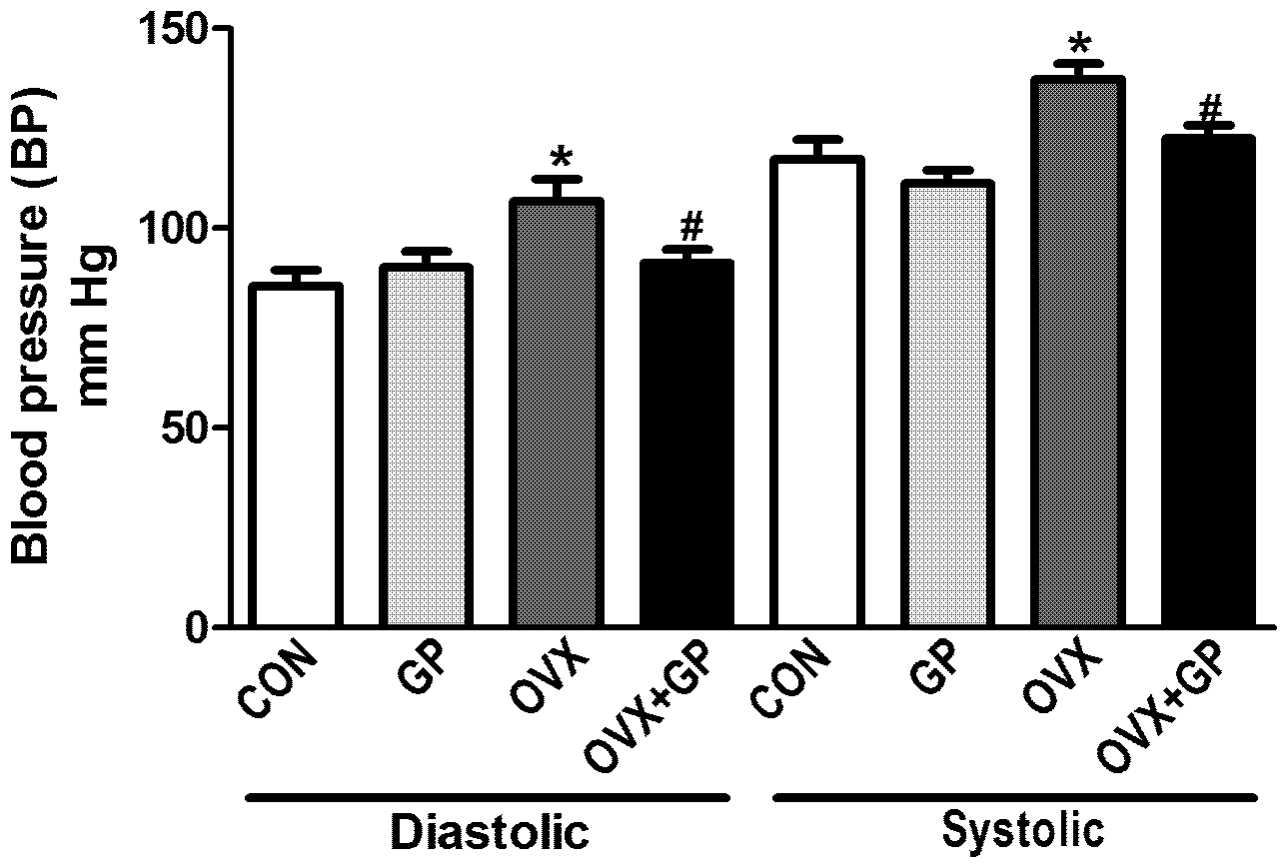
Examination of blood pressure in CON, GP, OVX and OVX+GP rats. *Left panel:* Diastolic blood pressure; *Right panel:* systolic blood pressure, *P*<0.05, (*) significantly different from control and GP and (#) significantly different from OVX. Bars are means ± SEM, *n* = 8–10 rats/group. Group designations: CON (control; sham operated rats provided with tap water for drinking), GP (grape powder: sham operated rats provided with GP treated tap water for drinking), OVX (ovariectomized: rats subjected to surgical removal of ovaries and provided with tap water for drinking), OVX+GP (rats subjected to surgical removal of ovaries and provided with GP treated tap water for drinking).

Systolic and diastolic blood pressure of OVX (systolic: 133±4 mm Hg, diastolic: 112±4 mm Hg) rats showed a significant increase as compared to CON (systolic: 120±3 mm-Hg, diastolic: 83±3 mm Hg) and GP (systolic: 114±2 mm Hg, diastolic: 86±3 mm Hg) rats, while OVX+GP (systolic: 122±2 mm Hg, diastolic: 86±2 mm Hg) rats showed blood pressure recordings similar to that of the controls **(** **Fig. 7** **)**.

### Assessment of GLO1, Mn-SOD and Cu-Zn SOD protein expression

**Figure 8:**
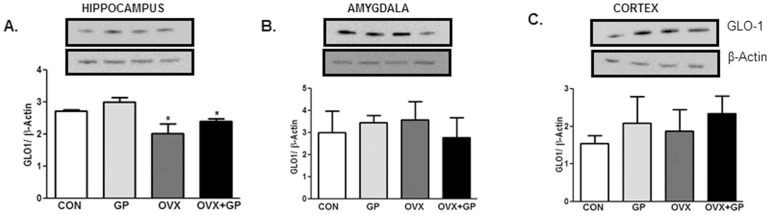
The levels of GLO-1 protein in the hippocampus, amygdala and cortex of CON, GP, OVX and OVX+GP rats. Protein levels of GLO-1 (A-C) were determined by western blotting. Upper panels in (A-C) are representative blots for GLO-1 and protein loading control β-actin. Bar graphs are ratios of respective protein/β-actin. Bars are means ± SEM, *n* = 3 rats/group, *P*<0.05, (*) significantly different from control and GP. Group designations: CON (control; sham operated rats provided with tap water for drinking), GP (grape powder: sham operated rats provided with GP treated tap water for drinking), OVX (ovariectomized: rats subjected to surgical removal of ovaries and provided with tap water for drinking), OVX+GP (rats subjected to surgical removal of ovaries and provided with GP treated tap water for drinking).

Protein expression levels of glyoxalase (GLO)-1 **(** **Fig.8A-C** **)**, Mn-superoxide dismutase (SOD) and Cu-Zn SOD (data not shown) were examined in the hippocampus, amygdala and cortex. While Mn-SOD and Cu-Zn SOD protein expression remained unchanged in all groups (data not shown), GLO-1 protein expression levels decreased in the hippocampus **(** **Fig.8A** **)** but not in the amygdala **(** **Fig.8B** **)**, or cortex **(** **Fig.8C** **)** of OVX rats. GP treatment failed to prevent the decrease in GLO-1.

## Discussion

In the present study, we provide evidence to suggest that estrogen depletion via ovariectomization increased anxiety-like behavior, caused memory impairment and increased blood pressure in female Wistar rats. Moreover, ovariectomization also led to increased oxidative stress in these rats. Interestingly, treatment of OVX rats with grape powder for 3 weeks was marked by improvement in anxiety-like behavior and learning-memory function, restoration of both systolic and diastolic blood pressure as well as reduction in oxidative stress. Earlier, relevant to these observations, protective effect of grape powder also was observed in oxidative stress-induced anxiety-like behavior, memory impairment and hypertension in a rodent model of oxidative stress [22]. The preventive effects on anxiety-like behavior, memory impairment and hypertension were attributed to the antioxidant properties of grape powder.

Preventive effects of drugs mimicking antioxidant effects have been reported on attenuation of high blood pressure [33], [39] and prevention of heightened anxiety-like behavior [33], [40], [41]. Oxidative stress was considered causal to these behaviors. While all of the above observations including our own [32]–[34] are quite interesting, of note is the fact that the aforementioned studies were conducted using pharmacological models utilizing pro-oxidant drugs as inducers of oxidative stress and synthetic compounds as antioxidants. Herein, we tested the role of oxidative stress in various impairments caused by estrogen deficiency and the antioxidant effect of grape powder in a more physiologically relevant model where potentially confounding effects of drugs are minimized. Actually estrogen depletion during menopause is reported to be associated with increased oxidative stress [7]–[9], a result of an imbalance between production and removal of reactive oxygen species via the antioxidant response system [10]. In fact, abnormalities in the redox system similar to those observed during menopause have been modeled using rodent ovariectomy models [23]. It is also known that ovariectomy can impair brain redox profile increasing oxidative stress [42]–[44].

These observations are in agreement with present data, which show elevated indices of oxidative stress including 8-isoprostane in the plasma and urine, protein carbonylation and nitrotyrosinylation in the hippocampus in response to ovariectomy and reversal of elevated oxidative stress levels upon GP treatment. Moreover, selective alteration in the levels of GLO-1, previously reported to be involved in oxidative stress mediated anxiety [45]–[47] but not in Cu-Zn SOD and Mn SOD levels is interesting. GLO-1, which is part of the cytosolic glyoxalase system, is reported to be activated upon inflammation and cytotoxicity [48]. Perhaps, OVX-mediated inflammation activates glyoxalase system which supersedes Cu-Zn SOD and Mn SOD mediated mitochondrial antioxidant defense mechanism(s). Furthermore, the hippocampus seems to be the brain region, which is most susceptible to ovariectomy-induced estrogen depletion, in terms of generation of oxidative stress as well as responsiveness to GP treatment. This region is likely to be important for modulation via nutritional intervention, as we did not observe any significant changes upon GP treatment in the amygdala or the cortex. Relevant to this, dentate gyrus (DG) is considered a critical part of the hippocampal formation responsible for pattern separation. DG is also a well-known area of the brain where neurogenesis occurs [49]. Thus, we investigated the effect of ovariectomy and/or grape powder treatment on DG granuler cells and observed an impaired LTP, a cellular correlate of learning and memory in OVX rats which was prevented with GP treatment.

The reason why hippocampus seems most susceptible to oxidative stress and consequently amenable to a nutritional intervention is premature to predict at this time. However, some insights can be drawn from previous knowledge. Involvement of amygdala, cortex and hippocampus in anxiety disorders [50], [51], central control of blood pressure [52], [53] as well as cognition [54] is known. Perhaps, ovariectomy-induced estrogen deficiency leads to activation of gamma amino butyric acid projections that transmit anxiety-related information from the amygdala to various centers in the brainstem [52], [53] and from there primary noradrenergic projections connect to the hippocampus, leading to specific biochemical alterations in the brain that ultimately affect behavior.

Furthermore, the association between estrogen depletion (menopause) and oxidative stress, mood disorders, hypertension and cognition also are linked with altered redox profile [55], [56]. Moreover, the antioxidant effect of grape powder polyphenols [57] have been reported on cognition, anxiety [57]–[59] and hypertension [60]. Therefore, it is reasonable to suggest that oxidative stress is caused by depletion of estrogen levels, as observed from elevated indices of oxidative stress in whole body parameters as well as in the brain of OVX samples. In the present study, oxidative stress is likely to be responsible for elevated anxiety-like behavior, learning-memory impairment and hypertension in OVX rats and grape powder, perhaps via its antioxidant effect, prevents these defects. This seems reasonable considering reported antioxidant [61], anti-anxiety [26], pro-cognitive [2] and anti-hypertensive _ENREF_4 [4] effects of estrogen. Our observations are important considering the high level of comorbidity reported between anxiety, hypertension and learning-memory impairment [62]–[67].

Therefore, we speculate that grape powder with its antioxidant properties has estrogen mimicking effects. This interesting proposition if proved accurate would have great therapeutic benefit for menopausal women who suffer from the highly prevalent comorbidity of anxiety, cognitive impairment and hypertension.
